# Varicose Hemorrhage from the Cervical Zone of the Uterus, Complicating Labor

**Published:** 1873-07

**Authors:** Gustavus C. P. Murray

**Affiliations:** Obstetric Physician to the Great Northern Hospital, late Senior Physician to the British Lying-in Hospital, and Vice-President of the Obstetrical Society of London


*Varicose Hemorrhage from the Cervical Zone of the Uterus, Complicating Labor.* By GUSTAVUS C. P. MURRAY, M.D., Obstetric Physician to the Great Northern Hospital, late Senior Physician to the British Lying-in Hospital, and Vice-President of the Obstetrical Society of London.

The rarity of cases of this kind is, I think, sufficiently shown by the little, if any, mention that is made of the subject in most of the published works on Obstetrics.

Dr. M'Clintock in his "Clinical Memoirs on the Diseases of Women," draws attention to what he terms *Uterine Hematocele*, and gives the history of two cases, both of which I shall hereafter recite, to show the points of resemblance and difference between them and those I am about to describe.

Mrs. B., aged twenty-seven, first labor, full term of gestation. In a weak, feeble state of health, without much stamina, and of a nervous temperament. She was about to retire to bed, when, with little or no labor warning, she felt that blood was running from her. Not being able to obtain the services of her engaged medical attendant, her husband requested Dr. Kirby to see her. The summons was quickly obeyed, and on his reaching the house, he found the patient in bed, very weak and fainting, owing to the loss of blood that had taken place, and which was still going on. On making an examination he felt what resembled the edge of the placenta, rather high up, and without loss of time the vagina was carefully plugged, and all further bleeding arrested. The os uteri was only partially dilated, and the liquor amnii had not escaped. It being suggested that a second opinion should be obtained, I had the pleasure of meeting Dr. Kirby.

Between the time of being sent for and arriving, the patient had lost little or no blood, and had not experienced any decided pains. After consultation, I determined on removing the plugs to see if delivery was practicable, and found the os uteri fully dilated, the membranes tense, and very little hemorrhage going on.

I passed my finger as high as possible within and around the cervix, but could not pronounce what was felt to be any portion of placenta. A full dose of ergot was given, the membranes were ruptured, and the labor progressed quickly and terminated favorably. The child was born alive, and the placenta expelled shortly afterwards without further hemorrhage; but we had some difficulty in keeping up the patient's strength and heat of body. Having failed to recognize the case as an ordinary one of placenta prævia, or accidental hemorrhage, we were left in doubt as to the source of bleeding, though the feature of the substance felt naturally suggested the presence of placental tissue.

I heard no more of this patient until thirteen months afterwards, when I was again sent for by Dr. Kirby to see her. She was now in labor with her second child, and, as on the previous occasion, hemorrhage had again set in as freely as it had done in her first confinement, and without there being any appreciable pain—in fact, exactly as before. On arrival, I found that Dr. Kirby had thoroughly plugged the vagina, administered nourishment, and given a fair amount of stimulant. Her general state of health had considerably improved since her previous confinement, and therefore she was now better able to bear the loss of blood.

After waiting some little time by the bedside of the patient, we observed a small *continuous stream* of dark-colored blood passing from the vagina. There were also some slight pains, and the bleeding seemed to increase somewhat at the commencement of them. After removing the plugs, I instituted a careful examination, and on reaching the os uteri, I found it dilated to the size of a crown, the membranes unruptured, and the fœtal head presenting. The cervix felt *large* and *pulpy* at its posterior aspect, and was rather low down in the vagina.

Passing my finger as far as possible within the uterus, I carried it well round the circumference of the cervix, but could not meet with the least trace of placenta. I however felt at one spot, corresponding to the external portion beforementioned as large and pulpy, a swelling rugose in character, with its edges raised and thickened. Two fingers distinctly passed over and beyond this enlargement, and reached true smooth uterine tissue on all sides. This fact, with that of not being able to detach any portion of the mass from the uterine wall, put the possibility of its being placenta out of the question.

On pressure with the fingers the swelling yielded, and became smaller, or reduced in size, and the bleeding was noticed to lessen or cease, returning again when the pressure was removed, and the hand when withdrawn was covered with venous blood. Dr. Kirby repeated all that I had done, and the same results were obtained. As labor was advancing, and the fetal head making fair pressure downwards, thereby materially staying the hemorrhage, we resolved to let nature take her course, assisted only by ergot and occasional restoratives. But we agreed to pass a plug dipped in a solution of iron, to the bleeding surface, if the hemorrhage again became excessive. There was, however, no necessity for this, and the labor terminated in the usual way, and favorably to both mother and child.

This second confinement demonstrated to us the cause of hemorrhage at the first labor, and it became clear and convincing, that these two early attacks of severe bleeding arose from the same source in both labors, and were due to an unusual varicose state of veins at the cervix uteri giving way during the commencement of labor. Under the circumstances attending the first labor, it was difficult, if not impossible, to arrive at anything like a correct diagnosis; but at the second confinement there was more time and opportunity afforded, and I was at the onset greatly assisted by calling to mind the absence of the diagnostic points of difference between unavoidable and accidental hemorrhage, and having before me symptoms differential in kind. The treatment adopted in both cases was simple and efficacious, requiring compression from below at the early stage of the labor, and subsequently the head in coming down gave the pressure from above, commanding or keeping in check the hemorrhage. It is important to mention that this lady, fourteen days after her second confinement, had a smart attack of uterine hemorrhage, requiring the immediate attention of Dr. Kirby, who succeeded in arresting it only after the employment of the appropriate remedies for such cases. Dr. M'Clintock says—"Let us now turn to that very rare variety of thrombus, in which the lip, or the lower part of the cervix of the womb, is the seat of extravasation. To all such cases I would restrict the term *Uterine Hematocele.*" He then gives the two following cases; the first was brought before the Dublin Obstetrical Society in 1850, by Dr. George Johnston, and was as follows:—"The patient, a robust countrywoman, aged thirty-five, in her seventh pregnancy, was delivered of a female child, evidently some time dead, after an easy labor of four hours, the breech having presented. The placenta was expelled in about ten minutes. No hemorrhage or untoward symptom of any kind supervened, and everything went on favorably for the first three days."

On the fourth day Dr. Johnston was sent for in a great hurry, owing to a violent attack of hemorrhage. On seeing the patient within four minutes from the first gush of blood, he "found her lying on her back, countenance perfectly blanched, and expressive of anxiety, which, with her neck, hands, and arms, was bathed in cold clammy perspiration. No pulse could be felt at the wrist, and the bed was inundated with blood, which was still flowing from the vagina." After using every effort to control the hemorrhage and recruit the patient's strength with some amount of success, the flooding recurred, and she fainted and rapidly sank. At the post-mortem the uterus was found well contracted. "On the left side of the cervix, about an inch from the os uteri, was observed a ragged, sloughy-looking opening, the edges of which were irregular, and of a black ash-gray color. This opening, which was large enough to admit two fingers easily, communicated with a cavity the size of a small orange. It seemed to be formed in the substance of the cervix, and its external wall was found to be the projecting tumor beforementioned, as seen from the outside." Dr. Montgomery, speaking of this case in the *Dublin Quarterly Journal*, 1851, says:—"A careful examination of the specimen convinced me that it was a case of thrombus, whose external envelope formed a thin layer of the uterine tissue, became gradually thinner, and finally ruptured."

Dr. M'Clintock's second case is taken from the writings of Mr. Roberton of Manchester. The patient was twenty-five years of age, third pregnancy, and delivered by turning in consequence of a hand presentation. The placenta was expelled without any unusual symptoms, and there was not much uterine discharge. On the eighth day after delivery she was seized with vomiting, and towards evening a sudden and very copious flooding came on; the quantity lost was estimated at two or three pounds. The hemorrhage appeared again after the lapse of two days to an alarming degree, and she died on the eighteenth day after delivery.

*Post-mortem.*—"Uterus not so much contracted as is usual. Near the neck on the left side, and between the folds of the broad ligament, there was some appearance of extravasation, and a sac partly filled with bloody pus was opened, which sac communicated by a large aperture with the general cavity of the uterus. On laying open the interior of the uterus there was the appearance of a deep excavation or ulceration, capable of readily admitting a finger or two, leading to this sac." Mr. Roberton "looked upon it as a partial rupture of the organ." "But," says Dr. M'Clintock, "there can be little doubt that this rupture of the uterine tissue was, in the first instance, the result of extravasation of blood into the muscular substance of the uterus."

Dr. Montgomery narrates the case of "a lady affected with varicose veins, which extended all up the lower extremities, and could be traced into the vagina, was delivered, after a natural and favorable labor, at midnight, but shortly afterwards a fearful rush of blood took place very unexpectedly, for the *uterus was well and firmly contracted.*" She became cold and pulseless, with no radial pulse for six hours. On examination, "in the situation of the anterior lip its substance felt as if broken up into a soft pulp, the consequence, as I believe, of the formation and rupture of a bloody tumor."

This patient ultimately completely recovered. In all the three cases given, the hemorrhage took place after labor, whereas in those I now bring forward it was present only during the first stage of labor, with a repetition in the second case, where it recurred at the end of fourteen days, and thus far resembling the cases quoted.

In the first two cases referred to, both ending fatally, the mischief in the uterine tissue must have extended deeper and covered a much larger surface than it had done in my cases and in Dr. Montgomery's; and doubtless, owing to the softening and disintegration of the muscular wall of the uterus at the site of the thrombus, perfect and *complete* contraction, most probably, could not take place, and the hemorrhage was not permanently arrested—whereas in the other cases, fortunately for our patients, the varicose condition being of a less severe character, the hemorrhage either ceased naturally or yielded to the treatment resorted to. Another point having probably much to do with the ultimate result of the case, is the situation of, and period of labor when, the rupture of the varix takes place. My cases and Dr. Montgomery's illustrate, as far as I know, the most favorable conditions of an unwished-for accident of the kind; and happily many conditions have to exist, or combine as it were, before this dangerous and unforeseen hemorrhage can take place either during or after labor.

The existence of a varicose state of the lower extremities during pregnancy, especially if extending upwards to the vulva, should, I think, put the obstetrician on his guard, not only as to the possibility of the occurrence of the hidden source of uterine hemorrhage we have been speaking of, but also as to the chance of the sudden rupture of the distended veins in and around the labia. In a work published in Paris in 1807, "Traite d'Accouchements, etc., par C. M. Gardien, M.D.," under the heading "Des Varices," he says, "Those tumors formed by the dilatation of some engorged veins show themselves most frequently about the eighth or ninth month of pregnancy. They are to be seen in the labia, vagina, and even at the *cervix uteri*. In this last situation they seriously interfere with labor, and great care is required on the part of the accoucheur, for fear of their rupturing and causing fatal hemorrhage.

Dr. Gardien adds that nothing should be omitted to prevent rupture, and recommends during the straining efforts of labor that the veins should be supported or strengthened by means of one or two fingers; and after delivery plugging is the most suitable method of keeping in check the hemorrhage produced by the rupture of a varix of the neck of the uterus.

Dr. M'Clintock, in his concluding remarks on the subject, after saying that it is one of extreme interest, even in a purely practical point of view, and having strong claims on our attention, states—"I have shown that it has a close bearing upon three of the forms of hemorrhage incidental to child-bed, viz., hemorrhage in the second stage of labor; hemorrhage immediately succeeding parturition; and secondary hemorrhage occurring some hours or days after delivery."

I would now, from the experience of the two cases I have brought before the profession, add a *fourth* and earlier stage at which this kind of hemorrhage may and has taken place—namely, during the *first* or *dilatation* stage of parturition.—*Obstetrical Journal.*

